# Assessment of SiO_2_ Nanotube Activity to Modify DL α-Tocopherol via ^1^O_2_ Generation Under Visible Light Irradiation

**DOI:** 10.3390/mi16070784

**Published:** 2025-06-30

**Authors:** Mihai Anastasescu, Radu Socoteanu, Veronica Bratan, Silviu Preda, Crina Anastasescu, Ioana Cătălina Gîfu, Cristina Lavinia Nistor, Rica Boscencu, Emilian Chifor, Catalin Negrila, Ion Bordeianu, Maria Zaharescu, Ioan Balint

**Affiliations:** 1 Institute of Physical Chemistry “Ilie Murgulescu”, 202 Splaiul Independentei, 060021 Bucharest, Romania; manastasescu@icf.ro (M.A.); psradu@yahoo.com (R.S.); vbratan@icf.ro (V.B.); predas01@yahoo.co.uk (S.P.); mzaharescu2004@yahoo.com (M.Z.); ibalint@icf.ro (I.B.); 2 National Research and Development Institute for Chemistry and Petrochemistry—ICECHIM, 202 Splaiul Independentei, 060021 Bucharest, Romania; lc_nistor@yahoo.com; 3 Faculty of Pharmacy, “Carol Davila” University of Medicine and Pharmacy, 6 Traian Vuia St., 020956 Bucharest, Romania; rboscencu@yahoo.com; 4 Faculty of Medicine, Ovidius University, Aleea Universitatii nr.1, 900470 Constanţa, Romania; ion_bordeianu@hotmail.com; 5 National Institute of Materials Physics, 405A Atomistilor St., 077125 Magurele, Romania; catalin.negrila@infim.ro

**Keywords:** SiO_2_ nano/micro tubes, photoactivity, reactive oxygen species, DL α-Tocopherol

## Abstract

This work investigates photoactive inorganic powders (SiO_2_, IrSiO_2_, and IrO_2_/IrSiO_2_) and their derivatives modified with metallated porphyrin, focusing on their ability to generate reactive oxygen species (ROS) under visible light exposure. The core material, SiO_2_, exhibits a tubular morphology and a high density of optically active defects. Modifiers such as metallic and iridium oxide nanoparticles, along with porphyrin, are employed to enhance light absorption and the generation of singlet oxygen (^1^O_2_) for potential biomedical applications. The time-dependent photogeneration of singlet oxygen is monitored using a Singlet Oxygen Green Sensor (SOSG), and its reactivity is evaluated in relation to DL α-Tocopherol through a spectrofluorimetric analysis. The photoactive materials, both before and after porphyrin modification, are characterized using Scanning Electron Microscopy (SEM), Atomic Force Microscopy (AFM), X-ray Diffraction (XRD), X-ray Fluorescence (XRF), UV–Vis Spectroscopy, X-ray Photoelectron Spectroscopy (XPS), N_2_ adsorption–desorption measurements, and zeta potential measurements.

## 1. Introduction

The latest technological and scientific advancements have brought significant benefits, but also inherent drawbacks such as pollution, aggressive maladies, and increased resistance among pathogens. Consequently, the development of valuable engineered biomaterials is essential for creating efficient technologies that support healthcare and human well-being, while remaining environmentally responsible. In this context, numerous studies have explored the role of reactive oxygen species (ROS)—including hydroxyl radicals (∙OH), superoxide anions (∙O_2_^−^), hydrogen peroxide (H_2_O_2_), and singlet oxygen (^1^O_2_)—in both synthetic and natural, and chemical and biological systems. ROS-based technologies have been increasingly applied in environmental remediation and fine chemical synthesis, where ROS formation is favored under suitable conditions (such as the presence of photocatalysts, O_2_, and sensitizers), promoting the mild or total oxidation of target substrates (organic or inorganic). In biological systems, whether exposed to light or not, ROS are mainly studied for their involvement in oxidative stress induced in the subjected cells. Many studies investigate the antimicrobial activity and theranostic potential of ROS-generating systems, including both endogenous/exogenous compounds and engineered materials, for improving health-related applications. Recent and valuable data, such as that by Younis et al. [1], highlight the role of inorganic nanomaterials as nano-photosensitizers (NPSs) in photodynamic therapy (PDT). These biocompatible nanomaterials include semiconductors (e.g., titanium dioxide and tungsten oxide), polymers, metals (e.g., gold and silver), carbon derivatives (graphene and graphitic carbon nitride), and silica. Moreover, the photochemical production of singlet oxygen and its behavior in biological systems, relevant to both light-assisted therapies and environmental remediation process, has been extensively reviewed by Pibiri et al. [2]. Valuable data on ROS generation and reactivity are often obtained by monitoring the time-course evolution of marker compounds. For instance, the fluorescence quenching of tocopherol upon its interaction with “in situ” generated singlet oxygen serves as a practical indicator. Tocopherol, a major component of Vitamin E, acts as an efficient antioxidant and singlet oxygen scavenger in natural systems [3]. It plays a crucial protective role in human cells by neutralizing singlet oxygen, which can otherwise damage cellular membrane [4] or alter DNA [5,6,7,8].

In general, the most extensively studied bioactive materials have focused on TiO_2_ derivatives [9], as TiO_2_ is a stable, biocompatible material capable of exhibiting antimicrobial activity and generating reactive oxygen species (ROS) upon light exposure. The present work aims to explore the potential of a novel functional material based on light-absorbing SiO_2_ nanotubes as an alternative to conventional TiO_2_ systems. Both TiO_2_ and SiO_2_ are biocompatible, non-toxic, and cost-effective oxides suitable for drug loading and controlled release applications [10], as well as for enzyme and biomarker immobilization of [11,12]. However, TiO_2_ loses its photoactivity and antimicrobial properties under visible light whereas light-absorbing SiO_2_ retains these functionalities. Moreover, the use of inorganic materials activated by light (solar or visible) to combat pathogens, including bacterial biofilms and viruses [13,14,15], has emerged as a promising research topic. The unusual photoactivity of tubular SiO_2_ was first reported by our group in 2009 [16], along with the catalytic behavior of Pt-modified tubular SiO_2_ which functioned as microreactors [17]. Subsequent studies have demonstrated the ability of optimized SiO_2_ to exhibit broad light absorption, extending from the UV to the visible range due to a high density of intra-band gap defects. This structural feature enables the photogeneration of singlet oxygen under simulated solar light exposure [18,19]. Additionally, correlations have been established between singlet oxygen generation on bare and modified SiO_2_ (functionalized with Au nanoparticles and Ru-based sensitizer) and the resulting antimicrobial activity against *Staphylococcus aureus* under solar and visible light irradiation [20].

The present research aims to carry out the following: (a) expand the range of previously reported singlet oxygen generators by modifying SiO_2_ nanotubes with iridium-based compounds and metallated porphyrin, specifically Zn(II)P; and (b) evaluate the reactivity of the ^1^O_2_ photogenerated by the resulting materials (SiO_2_, SiO_2_P, IrSiO_2_, IrSiO_2_P, IrO_2_IrSiO_2_, and IrO_2_IrSiO_2_P) under visible light irradiation using DL α-Tocopherol fluorescence quenching as an indicator. The application of iridium-based compounds for this purpose presents a significant degree of novelty, as IrO_x_-derived materials are primarily known for their use in capacitors and sensors, and as electrocatalysts in oxygen evolution reaction [21], and, more recently, due to their biocompatibility, as neural probes [22].

The advantages of the developed materials for biomedical applications include their biocompatibility and their ability to selectively photogenerate singlet oxygen under visible light irradiation, without producing hydroxyl radicals. This is particularly important, as hydroxyl radicals exhibit non-selective and aggressive oxidative behavior at the cellular level.

The present work introduces photoactive materials based on tubular SiO_2_ with potential biomedical applications, emphasizing their antimicrobial activity and capacity to photogenerate singlet oxygen (^1^O_2_), thereby enabling photodynamic therapeutic effects. Specifically, this study investigates both the cumulative photogeneration of singlet oxygen—by integrating conventional ^1^O_2_ generators such as porphyrins and metal/metal oxide nanoparticles (Ir, IrO_2_) with optically active, unconventional SiO_2_ nanotubes—and the reactivity of the photogenerated ^1^O_2_ toward DL α-Tocopherol assessed through a spectrofluorimetric analysis.

## 2. Materials and Methods

### 2.1. Material Synthesis

#### 2.1.1. SiO_2_ Nanotubes

The sol–gel synthesis of optically active SiO_2_ with tubular morphology has been reported earlier [18]. The main precursors used were the tetraorthosilicate (TEOS, 99%, Alfa Aesar, Ward Hill, MA, USA), DL tartaric acid (TA, Riedel de Häen, Seelze, Germany), and absolute ethanol (99.5% Merck, Darmstadt, Germany), following a molar ratio of 1 TEOS/0.035 TA/21.5 C_2_H_5_OH/18 H_2_O. Ammonia gas, bubbled from heated NH_4_OH solution (30% Roth), was added while the mixture was cooled to 0 °C. The resulting compounds were filtered, dried at 100 °C, and thermally treated in air at 500 °C, for 3 h.

#### 2.1.2. IrSiO_2_ Nanotubes

The one-pot synthesis of silica nanotubes containing iridium nanoparticles followed a similar procedure to that described above. Additionally, 0.02 g of Iridium pulv. (99.9%, Fluka, Buchs, Switzerland) was slowly added under gentle stirring.

#### 2.1.3. IrO_2_IrSiO_2_ Nanotubes

The pre-synthesized IrSiO_2_ nanotubes, previously calcined at 500 °C, were further modified with Chloroiridic (IV) acid (H_2_IrCl_6_∙6H_2_O, Fluka, 30–40% Ir) dissolved in absolute ethanol, and then thermally treated at 550 °C for 3 h.

#### 2.1.4. Porphyrin Synthesis

The synthesis of Zn(II)-5-(4-hydroxyphenyl-10, 15, 20-tris-(4-acetoxy-3-methoxyphenyl) porphyrin (hereafter denoted as P) has been previously described by Socoteanu et al. [23] and Boscencu et al. [24]. High-purity grade chemicals and solvents from Sigma-Aldrich and Merck were used.

#### 2.1.5. Preparation of SiO_2_P, IrSiO_2_P, and IrO_2_IrSiO_2_P 

To prepare the porphyrin-functionalized SiO_2_ derivatives with porphyrin: 0.0001 g of porphyrin was dispersed in 0.5 mL of absolute ethanol. A volume of 30 µL of this solution was used to impregnate 0.003 g of the pre-synthesized SiO_2_-based powders, followed by drying.

### 2.2. Singlet Oxygen Monitoring

The photogeneration of singlet oxygen was monitored using photoluminescence (PL) Spectroscopy on a Carry Eclipse fluorescence spectrometer (Agilent Technologies, Santa Clara, CA, USA) following the procedure reported in [19]. Photoactive powders (0.001 g) were mixed with a 5 mM methanolic solution of SOSG (Singlet Oxygen Sensor Green, Thermo Fisher Scientific, Waltham, MA, USA/Invitrogen, Carlsbad, CA, USA) and exposed to visible light irradiation (λ > 420 nm, Peccel solar simulator with cut-off filter). The SOSG-^1^O_2_ compound was detected via PL signal with an emission wavelength (λ_em_) of 530 nm and excitation wavelength (λ_ex_) of 480 nm, using excitation/emission slits of 2.5/2.5, at room temperature.

### 2.3. DL α-Tocopherol Fluorescence Quenching

A stock solution of DL alpha-Tocopherol (Glentham Life Science, Corsham, UK > 96%) was prepared by using 10 mg DL α-Tocopherol and 5 mL Methanol (AppliChem ITW Reagents, Darmstadt, Germany, 99.9%). For the fluorescence quenching experiments, 0.002 g of SiO_2_-based powders were suspended in 5 µL of this solution diluted in 3 mL methanol. The suspension was deposited in quartz cuvette and exposed to visible light for 5, 15, and 30 min. After 30 min of irradiation, 200 µL of D_2_O was added and the sample was further irradiated for an additional 30 min. The characteristic photoluminescence (PL) signal of tocopherol with a maximum at 327 nm (λ_ex_ = 290 nm) was monitored after each irradiation interval. Measurement parameters included excitation/emission slit widths of 2.5/2.5 nm and a scan rate of 120 nm/min.

### 2.4. DL α-Tocopherol Absorbance Quenching

In the absorbance quenching experiments, 0.002 g of SiO_2_-based powders were suspended in 5 µL of the stock solution and 3 mL methanol, then deposited into a quartz cuvette and exposed to visible light. UV–Vis spectra were recorded before and after 30 min of irradiation. Subsequently, 200 µL of D_2_O was added, and the sample was further irradiated for an additional 30 min. Spectral measurements were carried out using an Analytik Jena (Jena, Germany) Specord 200 Plus spectrophotometer.

### 2.5. Characterization Methods

The morphology of the samples was evaluated by SEM using an FEI Quanta 200 instrument (Eindhoven, The Netherlands) operated at an accelerating voltage of 30 kV. Images were acquired using a large field detector (LFD) in low-vacuum working mode.

AFM measurements were conducted in non-contact mode using an XE100 AFM system (Park Systems, Suwon, Republic of Korea), having flexure-guided, cross-talk-eliminated scanners. All AFM measurements were performed with PPP -NCHR tips (Nanosensors™, Neuchatel, Switzerland) with the following characteristics: <8 nm tip radius, ~125 μm length, ~30 μm mean width, thickness ~4 μm, ~42 N/m force constant, and ~330 kHz resonance frequency. AFM images were processed using the XEI program (version 1.8.0—Park Systems) for display purposes and surface roughness evaluation. Surface roughness was assessed using the root mean square roughness (R_q_), representing the standard deviation of the height value in the image and the peak-to-valley parameter (R_pv_) indicating the height difference between the lowest and the highest points. Representative line scans were also presented showing the surface profile of the scanned samples.

The diffuse reflectance UV–Vis spectra of the SiO_2_-based samples were recorded using a Perkin Elmer Lambda 35 spectrophotometer. The spectra were subsequently converted into absorption spectra using the Kubelka–Munk function.

X-ray diffraction (XRD) patterns of the investigated samples were obtained using a Rigaku Ultima IV multipurpose diffraction system (Rigaku Corp., Tokyo, Japan), under the following conditions: 30 mA and 40 kV, and room temperature. The 2θ range was scanned from 5 to 85°, with a step size of 0.02° and a scan rate 2°/min.

X-ray fluorescence (XRF) measurements were conducted to determine the elemental composition of the inorganic materials using a Rigaku ZSX Primus II spectrometer (Rigaku Corp., Tokyo, Japan), operating with wavelength-dispersive detection under vacuum. The chemical composition of the samples was determined based on Rigaku’s SQX 5.18 analytical software.

X-ray photoelectron spectroscopy (XPS) characterization was carried out using a SPECS spectrometer with a PHOIBOS (150) analyzer equipped with monochromatized Mg Kα (1487.6 eV) X-ray anode radiation source, operated at 300 W. The spectra were fitted using Voigt peak profiles and a linear or a Shirley background (depending on the peak shape), using the SDP v7.0 software (XPS International, Salem, OR, USA).

Nitrogen adsorption–desorption experiments were carried out on the calcined samples (500 °C), using a Quantachrome Nova 2200e apparatus (BET surface area, and pore volume and pore size distribution analyzer) produced by Quantachrome Instruments, Boynton Beach, FL, USA. The samples were vacuum-degassed at 350 °C for 4 h prior to the analysis. For the calculation of pore size distribution, Density Functional Theory (DFT) method was used, usually recommended for disordered materials. The textural properties were evaluated using the Nova Win 11.0 software.

Zeta potential (ξ) measurements were carried out using dynamic light scattering (DLS) on a Nano Zetasizer ZS (Malvern Instruments, Malvern, UK). The measurements were performed at room temperature.

## 3. Results and Discussions

### 3.1. SEM

**Figure 1:**
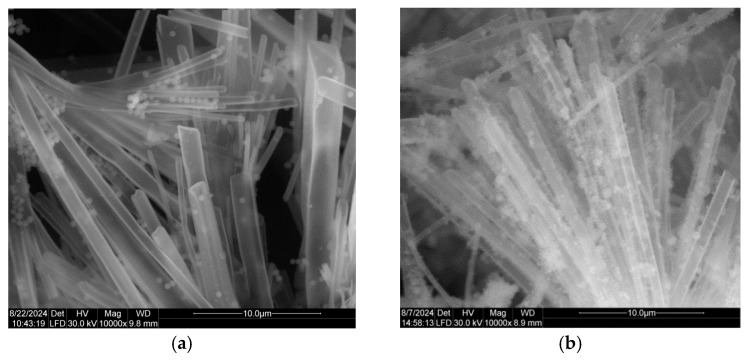

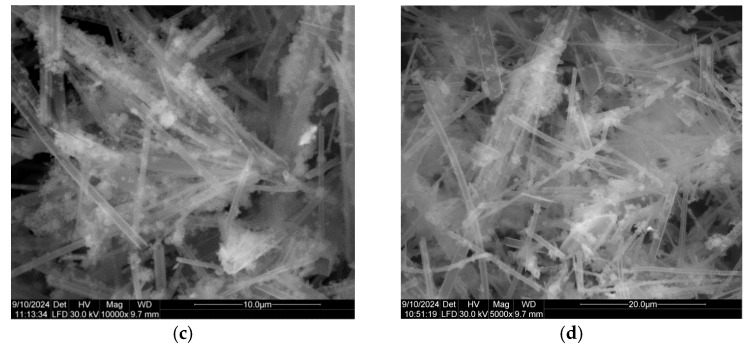
SEM images of SiO_2_- and iridium-modified SiO_2_ powders: SiO_2_ (**a**), IrSiO_2_ (**b**), and IrO_2_IrSiO_2_ at two magnifications (**c**,**d**).

The SEM images in Figure 1 show that the SiO_2_ nanotubes have lengths ranging from tens to hundreds of micrometers, and diameters on the orders of tens of nanometers. In addition to the tubular morphology, small spherical silica nanoparticles are also visible in all the SiO_2_-based sample which is consistent with our previously reported work [18] and other literature data. Iridium nanoparticles, added during the synthesis of SiO_2_, cannot be identified in the SEM image of the IrSiO_2_ sample. In the case of the IrO_2_IrSiO_2_ sample, the nanotubes appear less well-defined, likely due to the deposition of IrO_2_ on the surface of the pre-synthesized IrSiO_2_ nanotubes.

### 3.2. AFM

**Figure 2:**
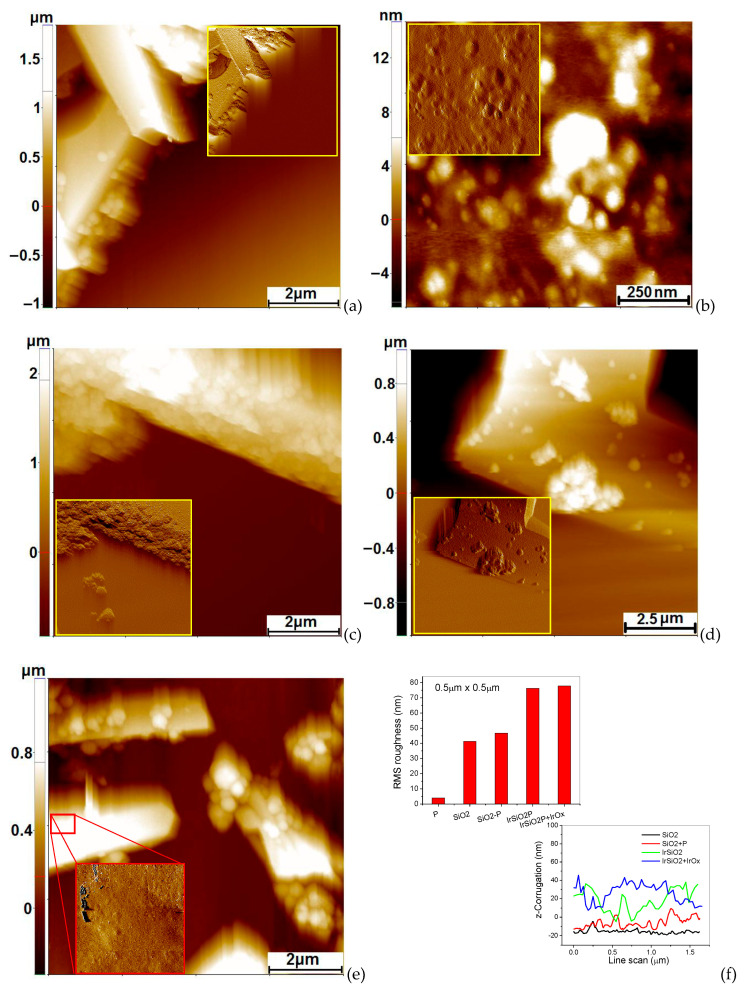
2D AFM images, topography, for the following: SiO_2_ sample with tubular morphology, (8 µm × 8 µm)—(**a**); metallated porphyrin, (1 µm × 1 µm)—(**b**); SiO_2_P sample with tubular morphology, (8 µm × 8 µm)—(**c**); IrSiO_2_P sample with tubular morphology, (10 µm × 10 µm)—(**d**); IrO_2_IrSiO_2_P sample with tubular morphology, (8 µm × 8 µm)—(**e**); and, in inset, Amplitude AFM images (at the same scales) with better contrast. Roughness histograms and random line scans collected along the tubes—(**f**).

AFM investigations (Figure 2) were performed by dispersing the prepared SiO_2_-based powders in ultra-pure water (Millipore Millie-Q system, Burlington, MA, USA). A drop of the resulting suspension was deposited onto a clean Si(100) substrate, allowed to dry at room temperature, and then placed in the AFM chamber. Figure 2a presents the AFM image of several adjacent SiO_2_ tubes (top-left corner) accompanied by spherical silica particles located at the edges of the tubes. The coexistence of tubular and spherical silica structures is a known outcome of the synthesis method [25]. The visible length of the SiO_2_ tubes is several micrometers, with diameters in the range of 1.1–1.2 µm.

The metallated porphyrin sample [24] was prepared using a similar method. Figure 1b shows the morphology of the porphyrin (P), consisting of randomly distributed quasi-spherical aggregates with diameters ranging from tens to hundreds of nanometers but with heights no exceeding 20 nm, most commonly around 10 nm [24]. Subsequently, the SiO_2_ powder modified with porphyrin (SiO_2_P) was analyzed, as shown in Figure 2c. The AFM image reveals that the surface of the imaged tube appears to be coated with aggregated particles ranging in diameter from several tens up to 200 nm. This suggests that the added porphyrin is deposited on the tube surface. However, the possible co-presence of silica particles cannot be excluded [25]. Figure 2d presents the AFM image of SiO_2_ tubes synthesized with iridium nanoparticles and subsequently modified with porphyrin (IrSiO_2_P). The results indicate that the addition of Ir nanoparticles during synthesis does not significantly alter the morphology of the silica tubes. However, porphyrin aggregates remain localized on the tube surface, as evidenced by the variations in particle aggregation compared to Figure 2c. Figure 2e presents the morphology of the IrO_2_IrSiO_2_P sample. In addition to the co-existence of spherical silica particles and porphyrin aggregates, smaller particles—presumably IrO_2_—are also observed, particularly in the inset of Figure 2e. This observation is supported by XRD and XRF analyses. AFM images recorded in Amplitude mode were superimposed onto the 2D topographic images in Figure 2, to enhance the morphological detail. For comparison, the RMS roughness was estimated from selected 0.5 µm × 0.5 µm areas of each sample. The SiO_2_ tube surface in Figure 2a exhibited an R_q_ of 41.2 nm and an R_pv_ of 204.8 nm. A similar area on sample P (Figure 2b) showed an R_q_ of 4.0 nm and an R_pv_ of 21.5 nm. For the SiO_2_P tube (Figure 2c), the roughness values were R_q_ = 46.8 nm and R_pv_ = 200.8 nm. In the IrSiO_2_P sample (Figure 2d), the roughness increased to R_q_ = 76.3 nm and R_pv_ = 260.7 nm. The inset analysis of Figure 2e, corresponding to the IrO_2_IrSiO_2_P sample (which displays small surface IrO_2_ particles 15–30 nm in diameter), revealed R_q_ = 78.6 nm and R_pv_ = 283.7 nm. The roughness histogram and representative line scans are shown in Figure 2f.

### 3.3. XRD and XRF

**Figure 3:**
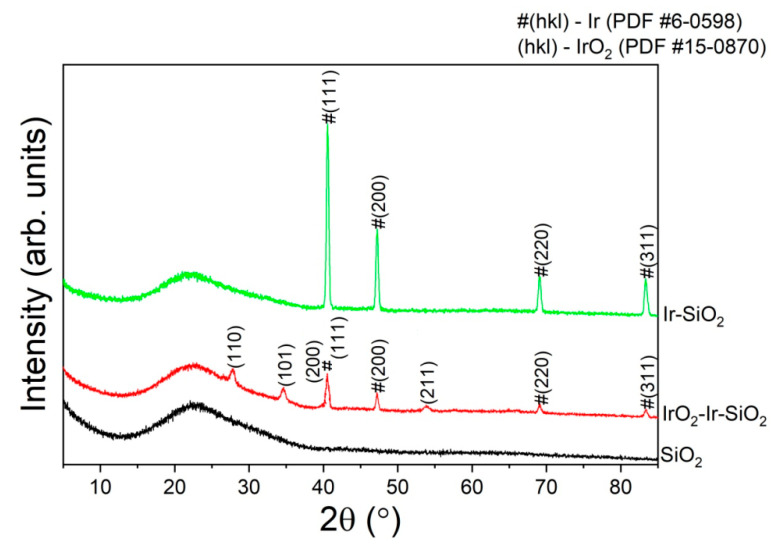
XRD patterns of unmodified and modified SiO_2_ nanotubes.

Figure 3 shows the amorphous structure characteristic of SiO_2_ nanotubes across all three samples. The presence of metallic Ir nanoparticles is confirmed in the IrSiO_2_ sample, while the IrO_2_IrSiO_2_ sample additionally displays the crystalline phase of IrO_2_. According to the XRF analysis, the Si:Ir ratio in the IrO_2_IrSiO_2_ sample is 82.7/3.3 (mass%).

### 3.4. UV–Vis Spectroscopy

**Figure 4:**
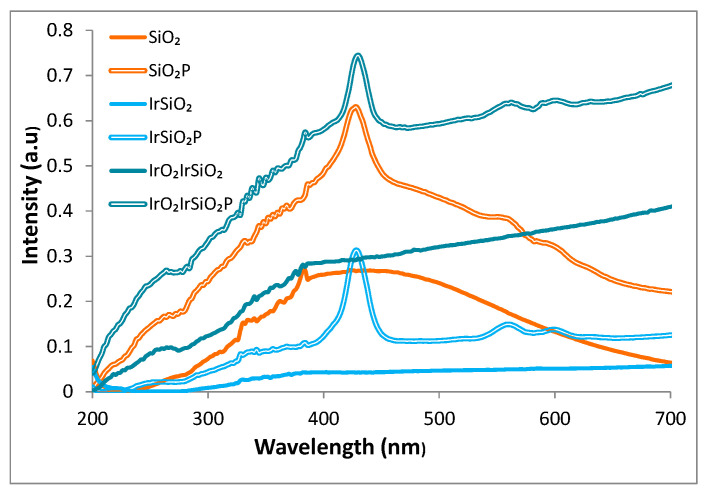
UV–Vis spectra of SiO_2_-based samples.

Figure 4 presents the UV–Vis absorption spectra of the SiO_2_, IrSiO_2_, and IrO_2_IrSiO_2_ samples, along with their hybrid derivatives containing porphyrin (SiO_2_P, IrSiO_2_P, and IrO_2_IrSiO_2_P), demonstrating the light absorption capacity across all samples.

Bare SiO_2_ displays a broad absorption band spanning from 300 to 700 nm, attributed to a high density of optically active defects [18,19]. The iridium-modified samples (IrSiO_2_, and IrO_2_IrSiO_2_) show a markedly different absorption profile, featuring a continuous plateau extending from the visible to the infrared region. The adding of porphyrin (P) to the inorganic matrices significantly enhances light harvesting—particularly in the visible region—evidenced by the characteristic absorption peaks centered at approximately 430 and 600 nm in all porphyrin-modified samples (SiO_2_P, IrSiO_2_P, and IrO_2_IrSiO_2_P).

### 3.5. XPS Spectroscopy

**Figure 5:**
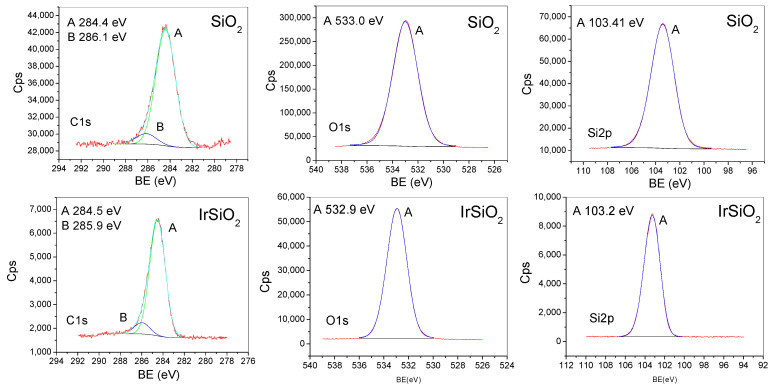

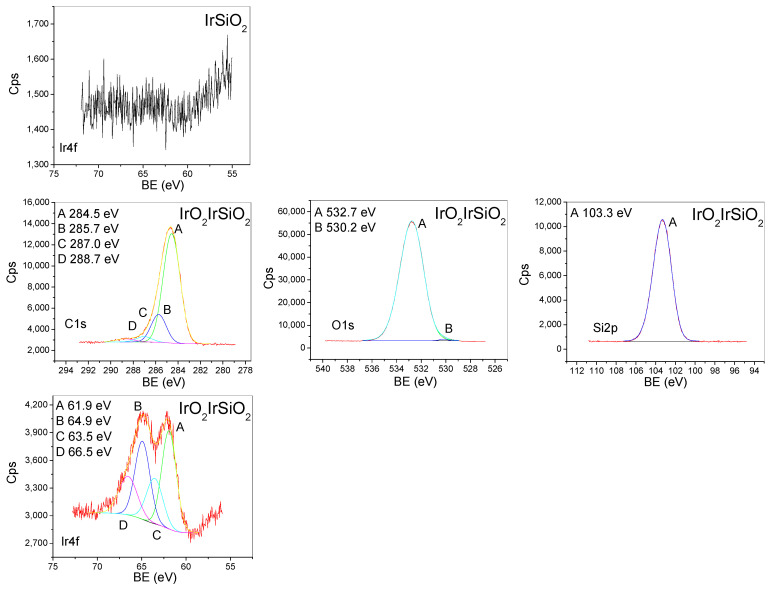
XPS high-resolution spectra (C1s, O1s, Si2p, and Ir4f lines) registered for the tubular SiO_2_, IrSiO_2_, and IrO_2_IrSiO_2_ samples.

The analysis of the XPS lines (Figure 5, Table 1) shows that the SiO_2_ and IrSiO_2_ samples have quite a similar surface composition, with no significant deviations from stoichiometry. The observed silicon and oxygen states are consistent with the presence of SiO_2_. 

Metallic iridium nanoparticles, added during the synthesis of the silica matrix, could not be detected on the IrSiO_2_ surface (likely due to being covered by silica). In contrast, the general spectrum of the IrO_2_IrSiO_2_ sample indicates the presence of iridium (0.1%). The A-B doublet may be assigned to Ir^4+^ (including C-D satellite features) or Ir^3+^, while the C-D doublet may be assigned to Ir^4+^. Given that the XRD analysis confirmed only the IrO_2_ phase, the dominant presence of Ir^4+^ is the most probable interpretation.

### 3.6. N_2_ Adsorption–Desorption Measurements

**Figure 6:**
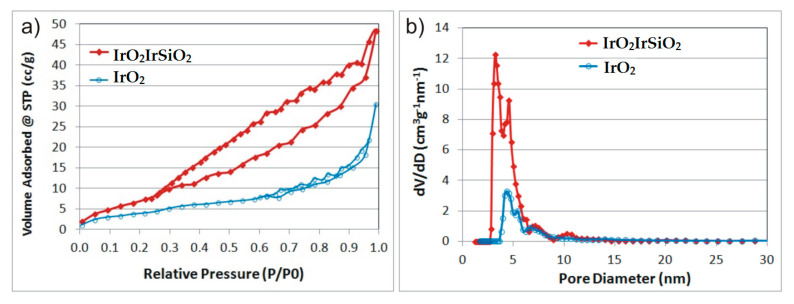
Textural analysis of samples IrO_2_IrSiO_2_ and IrSiO_2_: N_2_ adsorption–desorption isotherms (**a**); and DFT pore size distribution (**b**).

The N_2_ adsorption–desorption isotherms and corresponding pore size distribution diagrams for the samples of interest are presented in Figure 6. As previously reported [18], pure tubular silica exhibits a specific surface area in the range of 6–10 m^2^/g. Therefore, N_2_ adsorption–desorption measurements in this study were focused on the iridium-modified samples: IrSiO_2_ and IrO_2_IrSiO_2_. As shown in Figure 6, both IrSiO_2_ and IrO_2_IrSiO_2_ display type IV isotherms (according to the IPUAC classification), indicative of mesoporous materials. Additionally, both samples exhibit H3-type hysteresis loops, which are commonly associated with the presence of aggregates of plate-like particles that form slit-shaped pores.

Figure 6b shows polydisperse DFT pore size distributions for both materials, indicating the presence of pores of various sizes, ranging from 3 to 15 nm. The approximately two-fold increase in BET surface area (S_BET_), pore diameter (D_p_), and pore volume (V_p_) for the IrO_2_IrSiO_2_ sample compared to IrSiO_2_ (as shown in Table 2) confirms the higher porosity of IrO_2_IrSiO_2_. This is likely due to the post-synthesis deposition of IrO_2_.

### 3.7. Zeta Potential Measurements

**Figure 7:**
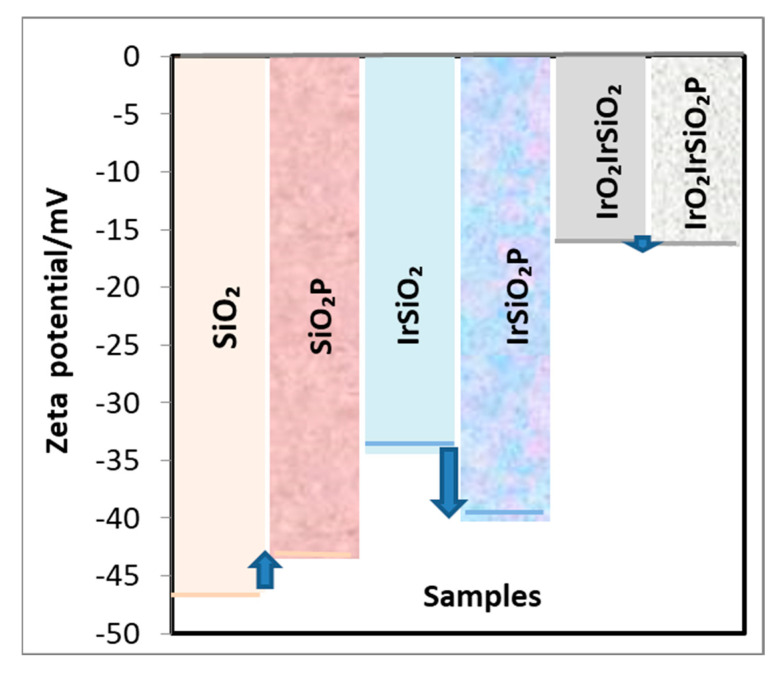
Zeta potential measurement.

Figure 7 displays the zeta potential values for all samples, showing negative surface charges. The incorporation of iridium nanoparticles into the SiO_2_ matrix (IrSiO_2_) causes a small shift in the zeta potential from −46.5 mV (pure silica) to more positive values of −34 mV. This shift becomes more pronounced upon the addition of IrO_2_, reaching the −16.1 mV value for the IrO_2_IrSiO_2_ sample. Figure 7 also highlights the effect of porphyrin loading to the inorganic matrices with blue arrows, showing, for SiO_2_, a slight positive shift to −43.5 mV. In contrast, in the iridium-modified sample (IrSiO_2_), the added porphyrin moves down the zeta potential value to −40.2 mV which differs significantly from the negligible change observed for the IrO_2_IrSiO_2_ sample (from −16.1 to −16.5 mV). These different trends may be attributed to the specific interaction between porphyrin and the surface of SiO_2_ both before and after the modification with Ir NPs and IrO_2_. In particular, the data suggest a tendency for the electrostatic stabilization of the unmodified SiO_2_ surface through porphyrin deposition.

### 3.8. ROS Photogeneration

**Figure 8:**
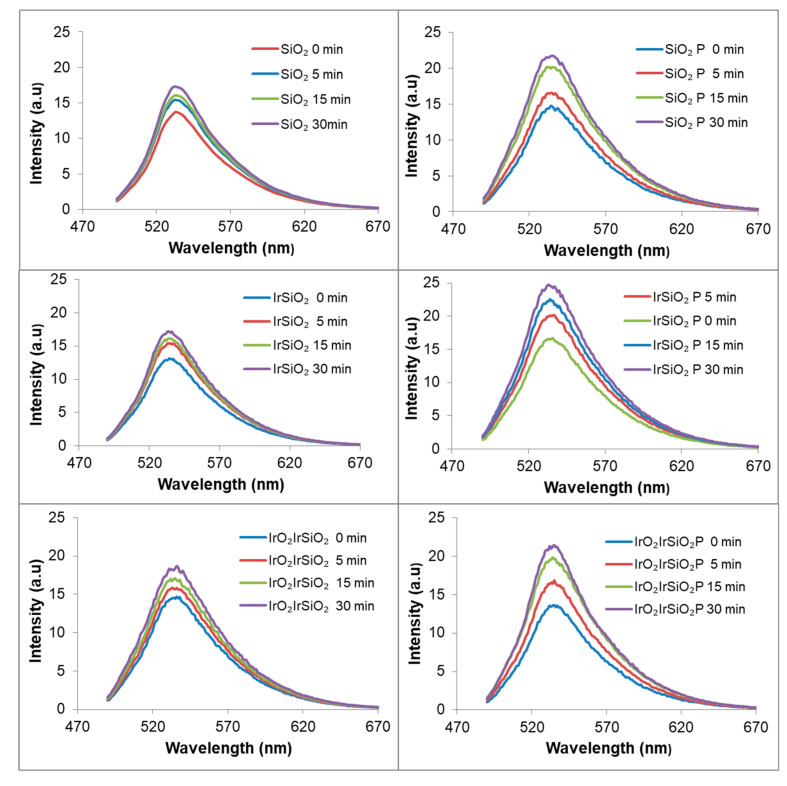
Time-course generation of singlet oxygen over SiO_2_-based nanotubes by using Singlet Oxygen Green Sensor (SOSG) under visible light irradiation.

The generation of reactive oxygen species (ROS)—specifically hydroxyl radicals (∙OH), superoxide anion (∙O_2_^−^), and singlet oxygen (^1^O_2_)—by the investigated materials under visible irradiation was assessed using previously established methods [19], employing coumarin, XTT sodium salt, and Singlet Oxygen Green Sensor (SOSG). No hydroxyl radicals or superoxide anions were detected; only singlet oxygen (^1^O_2_) was observed. Singlet oxygen generation was monitored via the detection of the endoperoxide product (SOSG-EP) resulting from the interaction of the SOSG with the singlet oxygen photogenerated by the SiO_2_-based samples under visible irradiation (λ > 420 nm). The resulting photoluminescence (PL) signal peaked at 535 nm, having increased with the irradiation time. As shown in Figure 8, all the inorganic samples (SiO_2_, IrSiO_2_, and IrO_2_IrSiO_2_) demonstrated the ability to generate singlet oxygen, with no significant differences among them.

Notably, the IrSiO_2_ sample did not exhibit improved singlet oxygen generation compared to bare SiO_2_, likely because the metallic Ir nanoparticles added during synthesis are embedded within the bulk of the nanotubes, rather than located on their surface. In contrast, the post-synthesis deposition of IrO_2_ nanoparticles on the surface of IrSiO_2_ nanotubes appears to enhance the overall singlet oxygen generation capacity of the resulting IrO_2_IrSiO_2_ material. The most important observation in Figure 8 is the significantly enhanced (nearly doubled) ability of the hybrid materials (i.e., SiO_2_-based matrices loaded with porphyrin) to produce singlet oxygen under visible light irradiation.

### 3.9. Tocopherol Decay-PL

**Figure 9:**
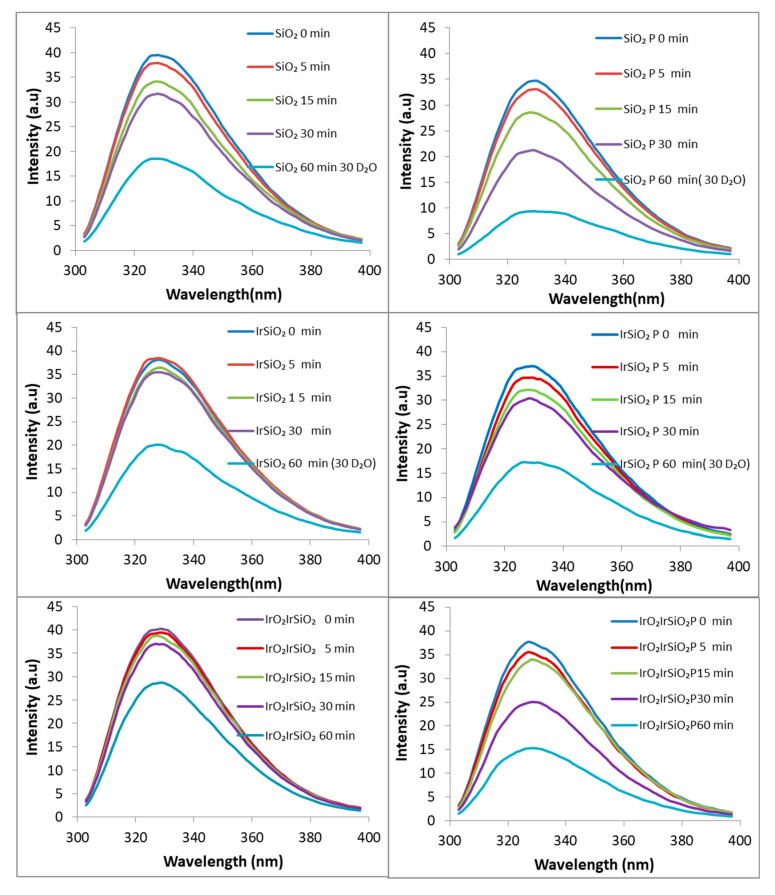
Decrease in PL signal of DL α-tocopherol in the presence of SiO_2_-based materials exposed to visible irradiation.

Alpha-tocopherol (α-T), a major component of Vitamin E, is commonly monitored using high-performance liquid chromatography (HPLC), often supported by complementary techniques [8,26,27]. As an innovative alternative, Demirkaya-Miloglu et al. [28] employed a spectrofluorimetric method to monitor α-T in human plasma and the pharmaceutical capsule using a standard ethanol solution of (α-T) with excitation and emission wavelengths of λ_ex_ = 291 nm and λ_em_ = 334 nm, respectively. Figure 9 illustrates the time-dependent decrease in the photoluminescence (PL) signal of DL α-tocopherol with a maximum at 327 nm (for λ_ex_ = 290 nm). The observed quenching is attributed to the singlet oxygen generated “in situ” under visible light irradiation (λ > 420 nm), by the SiO_2_, IrSiO_2_, and IrO_2_IrSiO_2_ samples and their hybrid porphyrin-functionalized derivatives.

The degradation of DL α-tocopherol in methanolic solution, in the presence of these photoactive powders after 5, 15, and 30 min of irradiation is more pronounced for the silica-based systems (both before and after porphyrin loading) compared to the iridium-modified materials. Furthermore, the addition of porphyrin to the inorganic matrices significantly enhances the PL decay of DL α-tocopherol in all three hybrids materials relative to their unmodified counterparts. For all tested systems, the addition of D_2_O drastically diminishes the PL signals of DL α-Tocopherol after 30 min of irradiation. Once more, the most significant decay of the investigated substrate is observed in the system containing the SiO_2_P sample. The DL α-Tocopherol decay shown in Figure 8 represents the combined contribution of several processes, including the photogeneration of singlet oxygen and its reactivity with DL α-Tocopherol. The interaction DL α-Tocopherol with the catalyst surface also impacts its degradation. From Figure 8, the IrO_2_IrSiO_2_ sample appears to exhibit a similar ability to generate singlet oxygen as the SiO_2_ sample. However, Figure 9 shows a smaller decrease in DL α-Tocopherol for the system containing IrO_2_IrSiO_2_. This discrepancy could also be related to Figure 7 which shows significant differences in the surface charges of the IrO_2_IrSiO_2_ and SiO_2_ samples. These differences may further influence the adsorption/desorption behavior of the DL α-Tocopherol and its reactivity with the singlet oxygen. In this study, the DL α-Tocopherol is used as a probe compound to evaluate the SiO_2_-based materials (both inorganic and porphyrin-functionalized) as singlet oxygen generators under visible light irradiation.

The photodecomposition of DL α-tocopherol under prolonged irradiation (240 min) with visible light (λ > 420 nm), in the absence of the investigated materials, was also performed and is presented in the Supplementary Materials as Figure S1. The PL signal of tocopherol was measured every 60 min, showing only a small decrease during the first 180 min, after which it remained stable. Comparative activity tests confirmed the intrinsic capacity of the samples to induce tocopherol degradation via singlet oxygen photo generation, enabling the identification of the most efficient materials.

The degradation of DL α-Tocopherol by “in situ” photogenerated singlet oxygen leads to various compounds, whose precise identification will require dedicated future research. Nonetheless, to establish valid correlations between the DL α-Tocopherol photodegradation and the structural characteristics of the singlet-oxygen-generating materials, the UV–Vis spectra of the systems were recorded and are provided in the Supplementary Materials as Figure S2. These spectra show not only a decrease in the DL α-Tocopherol absorbance peak with irradiation—consistent with the PL quenching shown in Figure 9—but also the emergence of new absorption peaks associated with reaction products, with slight differences in the product distribution.

The literature data on α-Tocopherol degradation reveal a wide range of products, depending on the experimental conditions. For example, De Vaugelade et al. [26] identified nine families of photolysis products under UV–Visible irradiation in acetonitrile. Miyazawa et al. [27] reported the formation of 8a hydroperoxy α-tocopherone (α-tocopherolhydroperoxide) following its interaction with singlet oxygen photogenerated by the methylene blue. Kaiser et al. [8], using HPLC with electrochemical detection, found tocopheryl quinone as a product of α-Tocopherol’s chemical reaction with singlet oxygen, along with other compounds. These authors also emphasized the physical quenching of ^1^O_2_ by α-Tocopherol, a process that increases with the solvent polarity (e.g., C_2_H_5_OH/CHC1_3_ vs C_2_H_5_OH/D_2_O), involving a charge-transfer mechanism. Similarly, Neely et al. [29] proposed that tocopherol quenches singlet oxygen via a charge-transfer mechanism, with efficiency increasing in more polar solvents. The resulting oxidation products include quinone and quinone-2,3-oxide. Kruk et al. [3] investigated the biochemistry of tocopherols in plants and their oxidation by ROS (including singlet oxygen) where a singlet oxygen addition leads to the formation of 8-hydroperoxychromanone.

**Figure 10:**
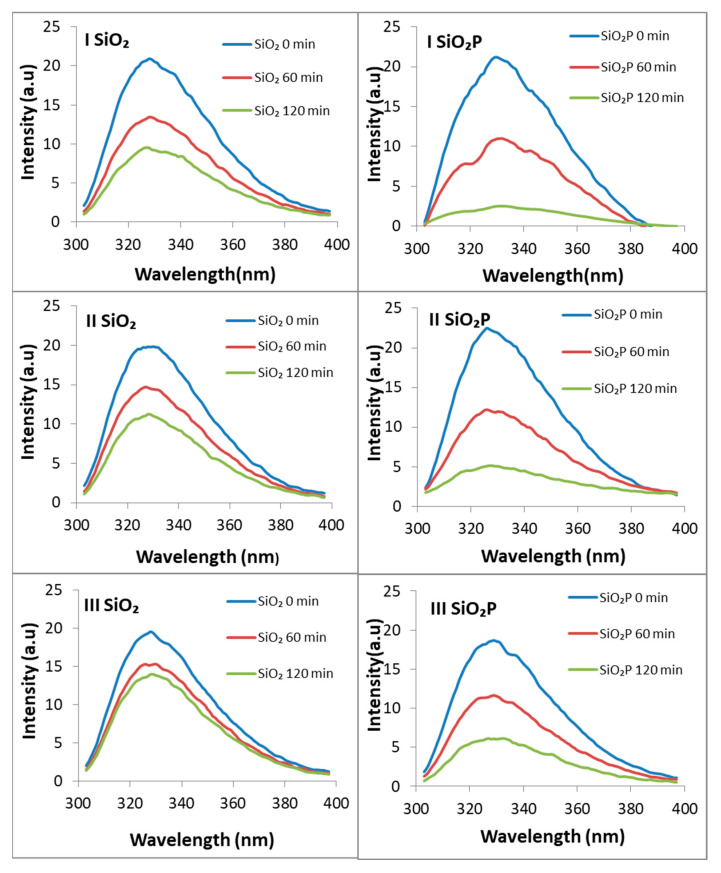
Stability and reusability of SiO_2_ and SiO_2_P samples exposed to visible irradiation.

To test the stability of the samples, the reaction time was extended to 120 min, without the use of D_2_O. The SiO_2_ and SiO_2_P samples exhibited the highest photoactivity during 120 min of light exposure, consistent with the data presented in Figure 9. Consequently, these two samples were subjected to two additional 120-min cycles. After each 120-min reaction cycle, the tocopherol solution was removed and the solid photocatalyst was washed with pure methanol to eliminate any adsorbed compounds. The decay of the DL tocopherol signal in the presence of these samples under visible irradiation across three successive reaction cycles (I, II, and III) is presented in Figure 10.

**Figure 11:**
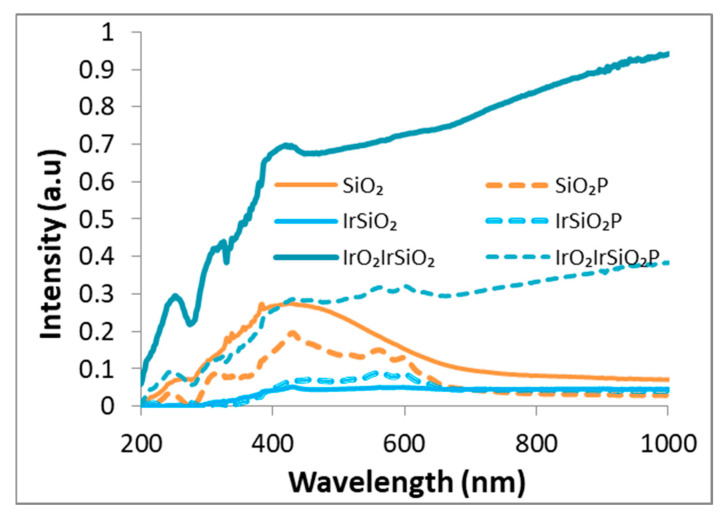
UV–Vis spectra of the spent samples.

For both SiO_2_ and SiO_2_P samples, tocopherol photodegradation is more pronounced during the first 60 min of each cycle; however, this trend is more evident for the unmodified SiO_2_ sample. In the case of porphyrin-modified SiO_2_, the difference between the first and last 60 min of reaction is diminished. Moreover, there is no substantial difference in activity between the first (I SiO_2_, and I SiO_2_P) and the second reaction cycle (II SiO_2_, and II SiO_2_P). However, in the third cycle, the PL signal of tocopherol shows a slight decrease for the SiO_2_ sample, whereas a continued decrease is observed for the SiO_2_P sample. This suggests that SiO_2_, and, especially, SiO_2_P, retains the ability to photogenerate reactive singlet oxygen throughout the entire 360 min of visible light irradiation. Due to their low activity, the IrO_2_ and IrO_2_IrSiO_2_ samples were tested only during a single 120-min reaction cycle (Cycle I), with their performance reported in the Supplementary Materials as Figure S3. The structural stability of the tested samples, both inorganic and porphyrin-functionalized, was evaluated using UV–Vis spectroscopy, as shown in Figure 11.

With reference to Figure 4, the absorptive properties of the spent SiO_2_, IrSiO_2_, and IrO_2_IrSiO_2_ samples (continuous lines) appear to be unaffected by the reaction medium. In contrast, their hybrid counterparts exhibit a reduced light absorption capacity compared to their initial state. The “fingerprint” of the porphyrin (located peaks at 430 and 600 nm) are significantly diminished in the recorded spectra. Although the SiO_2_P sample also demonstrates a considerable loss in light-harvesting capacity after three reaction cycles (I–III), the porphyrin’s characteristic peak at 430 nm remains clearly defined, proving an appropriate loading of the SiO_2_ matrix with porphyrin. This can explain its significant photoactivity in the third reaction cycle (Figure 10, III SiO_2_P).

Both experimental and literature data from the investigations underscore the relevance of the interaction between ^1^O_2_ and α-Tocopherol in natural and engineered systems. Accordingly, this study primarily explores the feasibility of using α-Tocopherol as a probe compound for monitoring “in situ” generated singlet oxygen. At the same time, it highlights the promising research opportunity in developing novel functional biomaterials capable of supporting mechanisms with biomedical and physiological significance, such as the controlled photogeneration of singlet oxygen for photodynamic therapy, antimicrobial applications, and interactions with natural substrates like tocopherol.

## 4. Conclusions

This paper highlights the development of new photoactive materials based on tubular SiO_2_ capable of generating singlet oxygen under visible light irradiation. The photogeneration of the singlet oxygen is enhanced by adding inorganic (Ir and IrO_2_ nanoparticles) and organic (porphyrin) modifiers. The observed modification of DL α-Tocopherol under visible light irradiation supports the potential of tubular SiO_2_, IrSiO_2_, IrO_2_IrSiO_2_, and their porphyrin-functionalized hybrids as effective singlet oxygen generators suitable for biomedical applications.

## Figures and Tables

**Table 1:** XPS line assignment.

Sample	C1s	O1s	Si2p	Ir4f
SiO_2_	Peak A (284.4 eV) C-C, C-H bonds Peak B (286.1 eV) C-O bonds (simple)	Peak A (533 eV) Oxygen in SiO_2_	Peak A (103.4 eV) Si^4+^ state (SiO_2_)	
IrSiO_2_	Peak A (284.5 eV) →C-C, C-H bonds Peak B (285.9 eV) C-O bonds (simple)	Peak A (532.9 eV) Oxygen in SiO_2_	Peak A (103.2 eV) Si^4+^ state (SiO_2_)	Ir 4f line is undefined
IrO_2_IrSiO_2_	Peak A (284.5 eV) →C-C, C-H Peak B (285.7 eV) C-O bonds (simple) Peak C (287 eV) O-C-O, C=O (double bonds) Peak D (288.7 eV) possible carbonate group	Peak A (532.7 eV) Oxygen in SiO_2_ Peak B (530.2 eV) Oxygen in iridium oxides	Peak A (103.3 eV) Si^4+^ state (SiO_2_)	Peak A (61.9 eV) Peak B (64.9 eV) Peak C (63.5 eV) Peak D (66.5 eV) (a) A-B doublet (Ir4f^7/2^, Ir4f^5/2^) → Ir^4+^(IrO_2_) C-D doublet—satellites (b) A-B doublet → Ir^3+^ C-D doublet → Ir^4+^

**Table 2:** Textural properties of the investigated samples.

Sample	S_BET_ (m^2^/g)	*V_T_ (cc/g)	V_P_ (cc/g)	D_p_ (nm)	S_DFT_ (m^2^/g)	V_P DFT_ (cc/g)	D_p DFT_ (nm)
IrO_2_IrSiO_2_	30.6	0.075	0.086	4.77	25.4	0.067	4.54
IrSiO_2_	15.7	0.047	0.059	2.95	8.3	0.032	4.34

V_T_ (To *V_T_—(Total Pore Volume); V_P_—(Pore Volume) BJH method desorption branch; D_p_—(Pore Diameter) BJH method desorption branch; V_P DFT_—(Pore Volume) DFT method; D_p DFT_—(Pore Diameter) DFT method.

## Data Availability

The reported data are included in the article.

## Supplementary Materials

The following supporting information can be downloaded at: https://www.mdpi.com/article/10.3390/mi16070784/s1, Figure S1: Decreasing of tocopherol absorbance in the presence of SiO_2_-based materials under visible irradiation. Figure S2: Decrease in tocopherol absorbance in the presence of SiO_2_—based materials under visible irradiation. Figure S3: Decrease in DL α tocopherol Pl signal under visible light exposure for 120 minutes in the presence of IrSiO_2_ and IrO_2_IrSiO_2_ samples.

## Abbreviations

The following abbreviations are used in this manuscript:

ROS	Reactive Oxygen Species
SOSG	Singlet Oxygen Green Sensor
SEM	Scanning Electron Microscopy
AFM	Atomic Force Microscopy
XRD	X-ray Diffraction
XRF	X-ray Fluorescence
XPS	X-ray Photoelectron Spectroscopy
