# Supplementary material for: Assessment of SiO2 Nanotube Activity to Modify DL α-Tocopherol via 1O2 Generation Under Visible Light Irradiation
Source: Micromachines (Basel). 2025 Jun 30;16(7):784. doi: 10.3390/mi16070784 (PMC12300390; doi:10.3390/mi16070784)
Supplement: Supplementary file 1 [file micromachines-16-00784-s001.zip › micromachines-3661862-supplementary.pdf]

Assessment of SiO_2 nanotubes activity to modify DL-α-Tocopherol via $^1O_2$ generation under visible light irradiation

Mihai Anastasescu, Radu Socoteanu, Veronica Bratan, Silviu Preda, Crina Anastasescu, Catalina Gifu, Cristina Lavinia Nistor, Rica Boscencu, Emilian Chifor, Catalin Negrila, Ion Bordeianu, Maria Zaharescu and Ioan Balint

I.

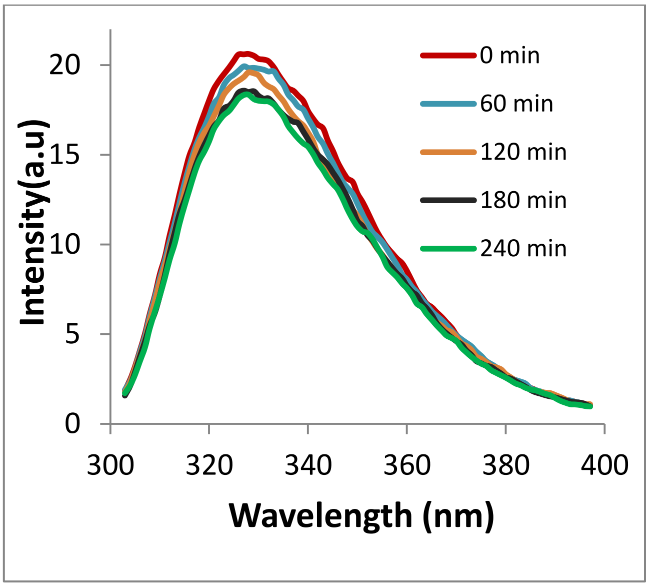

Figure S1 Decrease in tocopherol photoluminescence signal under visible exposure (λ > 420 nm) for 240 minutes.

A slight decay of tocopherol can be perceived in the first 60 minutes of light exposure, which continues up to 180 minutes, when it stops.

II.

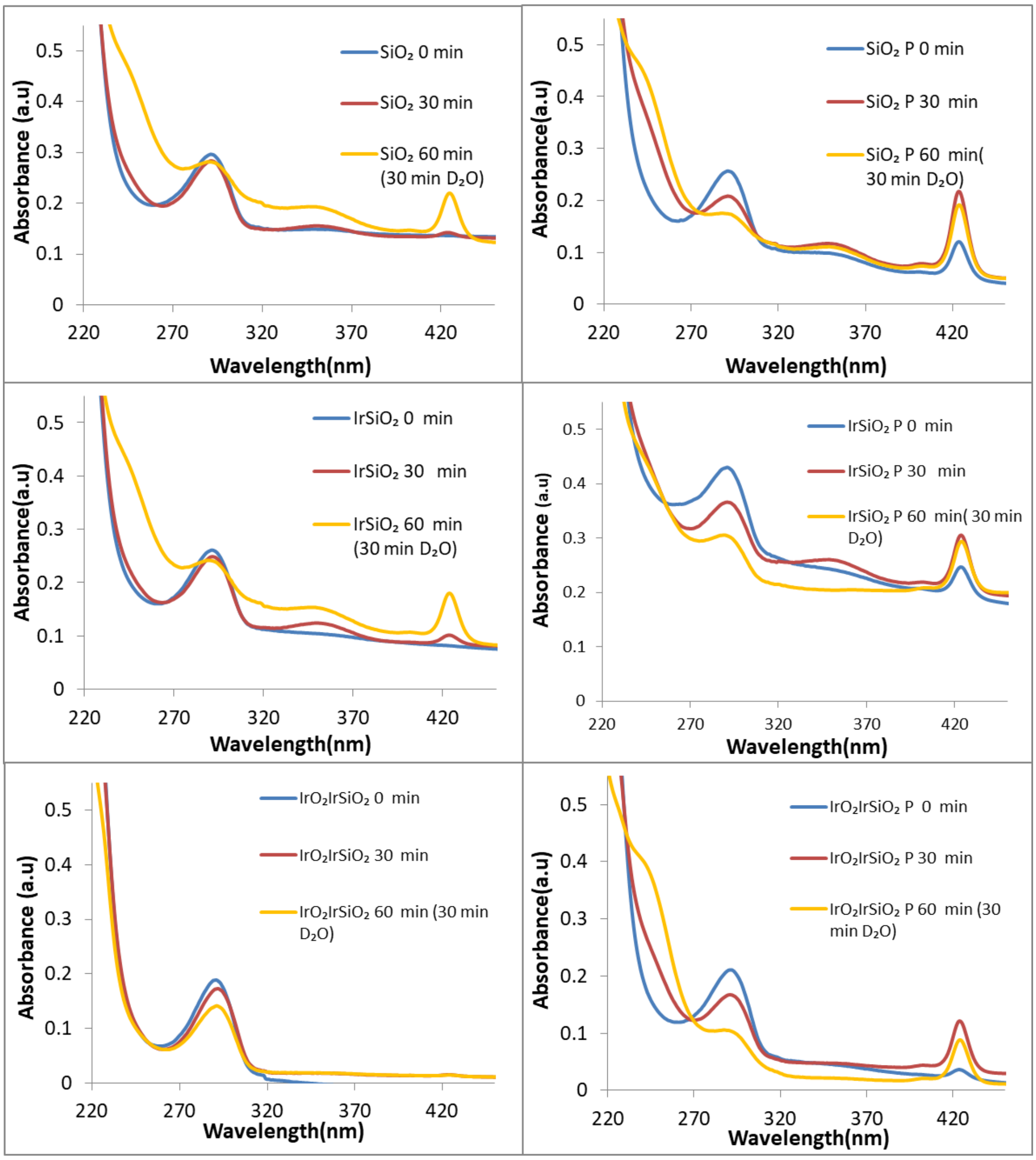

Figure S2 Decrease in tocopherol absorbance in the presence of SiO_2 - based materials under visible irradiation.

The DL α tocopherol absorbance is peaked at 290 nm, after 30 minutes of irradiation a small decrease is perceivable for all inorganic samples, the decay being strongly illustrated for the systems containing hybrid derivatives with porphyrin. Apart from the previously-mentioned peak, UV Vis spectra present a quite large absorption band around 340 nm and a well-defined peak centered at 417nm. D_2O adding and supplementary exposure for 30 minutes to visible irradiation brings for all the systems excepting that containing IrO_2IrSiO_2, a sharp decrease of the main peak from 290 nm and the appearance of a shoulder around 240 nm. The clearer decay of DL α-tocopherol absorbance and the best-represented reaction product distribution are registered for the system containing the SiO_2P sample.

III.

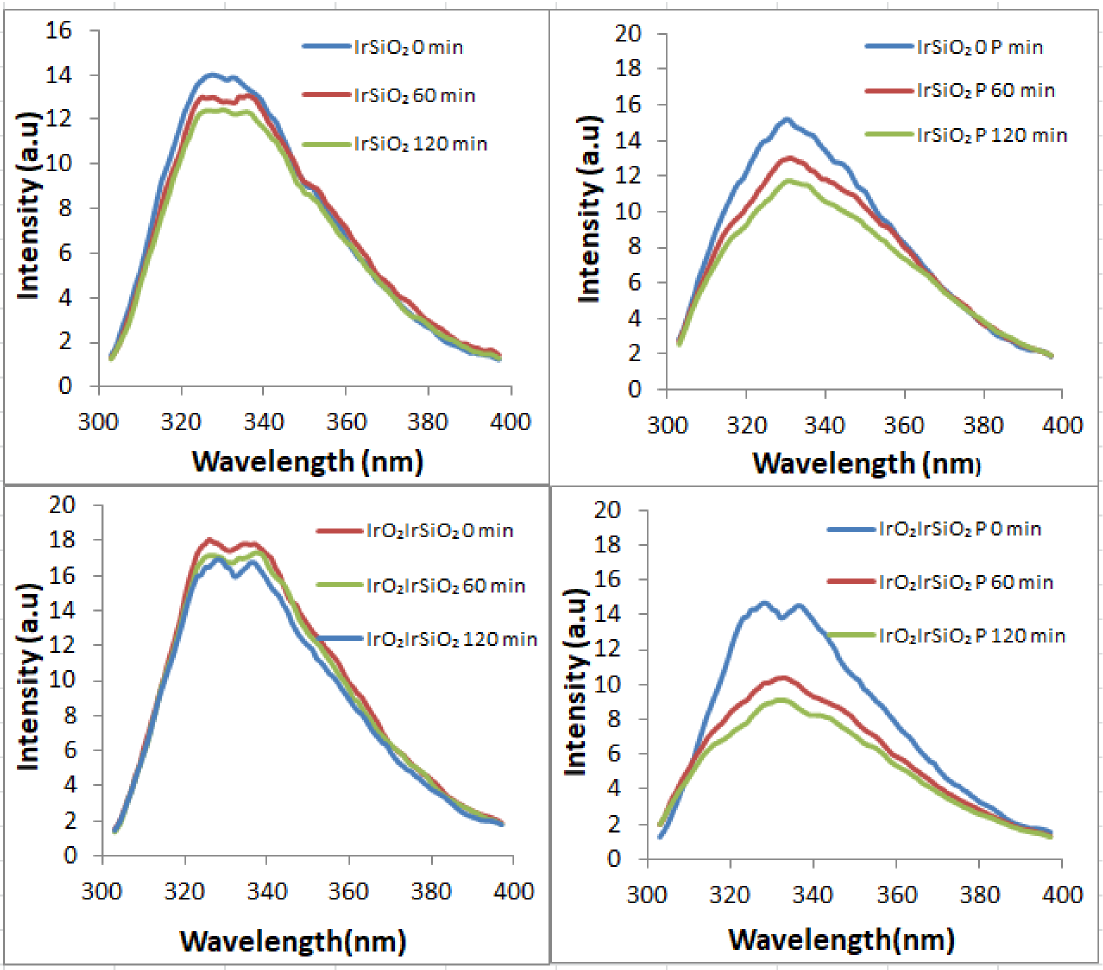

Figure S3. Decrease in DL *α* tocopherol Pl signal under visible light exposure for 120 minutes in the presence of $IrSiO_2$ and IrO_2IrSiO_2 samples

The decrease in PL signal of DL Tocopherol for the iridium-modified samples ($IrSiO_2$ and IrO_2IrSiO_2) after 120-minute reaction time is slight but in line with the results presented in Figure 9 from the manuscript during the first 30 minutes of reaction time. Their derivatives with porphyrin ($IrSiO_2P$ and IrO_2IrSiO_2P) emphasize a better photoactivity, most probably dependent on the loaded porphyrin onto the inorganic matrices. It can be assumed that, for longer irradiation time, the rate of the tocopherol decay is slower than in the first 30 minutes (Figure 9). One explanation could be the higher temperature of the reaction medium induced by the prolonged light exposure triggering the decrease in singlet oxygen photogeneration(favored by the low temperature).
